# Reply: Double vitrification and warming do not compromise the chance of live birth—a potential invalid conclusion

**DOI:** 10.1093/hropen/hoad050

**Published:** 2024-01-04

**Authors:** Sofia Makieva, Maike Katja Sachs, Min Xie, Ana Velasco Gil, Samia El-Hadad, Dimitrios Rafail Kalaitzopoulos, Ioannis Dedes, Ruth Stiller, Brigitte Leeners

**Affiliations:** Department of Reproductive Endocrinology, University Hospital Zurich, Zurich, Switzerland; Department of Reproductive Endocrinology, University Hospital Zurich, Zurich, Switzerland; Department of Reproductive Endocrinology, University Hospital Zurich, Zurich, Switzerland; Department of Reproductive Endocrinology, University Hospital Zurich, Zurich, Switzerland; Department of Reproductive Endocrinology, University Hospital Zurich, Zurich, Switzerland; Department of Gynaecology, University Hospital Zurich, Zurich, Switzerland; Department of Reproductive Endocrinology, University Hospital Zurich, Zurich, Switzerland; Department of Gynaecology, University Hospital Zurich, Zurich, Switzerland; Department of Gynaecology, University Hospital Zurich, Zurich, Switzerland; Department of Reproductive Endocrinology, University Hospital Zurich, Zurich, Switzerland; Department of Reproductive Endocrinology, University Hospital Zurich, Zurich, Switzerland

Sir,

We appreciate the opportunity to provide a response to the letter penned by Nanassy and esteemed colleagues in Germany (Nanassy *et al.*, 2023a), pertaining to our study (Makieva *et al.*, 2023). We performed a retrospective analysis of frozen single embryo transfers (SETs) showing that vitrifying embryos twice, first as zygotes and then as blastocysts, does not reduce the chance of live birth compared to one round of vitrification at the blastocyst stage. However, Nanassy *et al*. have also recently published a retrospective study demonstrating that live birth is reduced in the case of double vitrification (Nanassy *et al.*, 2023b). Hence, they contend that we should have conducted an alternative analysis akin to the one undertaken in their study.

Firstly we would like to address the overarching concern before delving into the specific points raised by Nanassy *et al*., namely, the strategy of our analysis. They do not appreciate that we included multiple embryo transfers (ETs) and not a single ET per patient and that we have utilized chi-square analysis instead of a multivariate analysis to account for the various biases they have posited. We reported the characteristics per cycle and per ET for both groups: vitrified once (single vitrification-warming: SVW) and double vitrified (double vitrification-warming: DVW), including the number of multiple ETs per group. Notably, our analysis did not reveal any statistically significant difference between these two groups. The only difference was the time of storage of cryopreserved embryos in the two groups. Nonetheless, it is evident from existing literature that this particular characteristic does not exert a discernible influence on live birth outcomes (Canosa *et al.*, 2022). Consequently, we deemed the groups to exhibit homogeneity. If we had identified the presence of confounding factors, we would have duly employed a multivariate analysis to account for such variables. Similar studies to ours have adopted chi-square tests to evaluate clinical outcomes associated with multiple rounds of vitrification (for example Kumasako *et al.*, 2009; Zheng *et al.*, 2017; De Vos *et al.*, 2020; Li *et al.*, 2021). It is worth noting that these studies also did not encompass a fresh cycle cohort, a point of contention raised by Nanassy *et al.* (2023a) in their letter. However, in alignment with the research scope defined for our study, we, akin to others, deemed the evaluation of fresh cycle outcomes to be beyond its purview. Our primary objective was to investigate the impact of multiple rounds of vitrification in contrast to a single round of vitrification.

A second facet of concern posited by Nanassy *et al.* (2023a) in their letter pertains to the potential presence of a poor prognosis bias in our study. They allude to various criteria that could signify a poor prognosis, including a freeze-all cycle, multiple transfers, utilization of Day 6 blastocyst transfers or a prioritization strategy for zygotes in fresh or warming cycles. We would like to refute these allegations point by point.

With regard to the freeze-all indication, we must clarify that a significant proportion of our cryopreserved zygotes in the cohort stem from routine laboratory practices, rather than freeze-all associated with poor prognosis. Our laboratory adheres to a particular practice of freezing zygotes, thereby offering flexibility in performing transfers at both the cleavage and blastocyst stages during subsequent warming cycles. We neither prioritize nor discriminate among zygotes scored between Z1 to Z3 grade in fresh or warming cycles, as the predictive value of pronuclear scoring in relation to clinical outcomes remains equivocal (Nicoli *et al.*, 2013). Poor quality zygotes, such as those rated as Z4, rarely progress to the blastocyst stage and in our study, exclusively transfers of blastocysts with a minimum quality grade of BB were included. Our particular routine was discussed during the peer review process for our article (Makieva *et al.*, 2023), where the reviewers suggested to report blastulation rates per the zygotes thawed, regardless—and not per all zygotes in a cohort.

Regarding the Day 5 and Day 6 blastocysts and their potential for live birth, practical considerations, laboratory routines, and numerous other factors can lead to the cryopreservation of blastocysts on Day 6. Furthermore, the same blastocyst may be graded as a 4AA on Day 5 and a 5AA on Day 6, when it is eventually cryopreserved. Thus, the notion that Day 6 blastocysts are universally indicative of slower development can be a misconception. In fact, emerging studies suggest that Day 6 blastocysts of superior quality may outperform Day 5 counterparts (Shi *et al.*, 2022; Wu *et al.*, 2023), while other research shows that clinical outcomes are indistinguishable between Day 5 and Day 6 blastocysts exhibiting comparable morphological quality (Chen *et al.*, 2023). In our study, we include blastocysts of a minimum BB quality, as we do not freeze poor prognosis blastocysts.

Finally, the prospect of multiple transfers as an indicator of poor prognosis and a source of bias has been diligently addressed in our study. Cases where patients underwent both SVW and DVW were meticulously excluded from our analysis. The distribution of the number of embryo transfers per patient in the SVW and DVW groups revealed no significant disparities, thereby substantiating the equivalency of our sample populations (*P *=* *0.063).

In our discussion, we emphasize that there are more studies on the topic that show no impact of multiple rounds of vitrification on clinical outcomes. As we accumulate more evidence, a throughout meta-analysis of previous, ours and Nannassy *et al*.’s works will shed light on the topic. Until such a synthesis is conducted, the majority of IVF clinics may continue to employ multiple rounds of vitrification with confidence, given that this practice appears to yield neonatal outcomes that are, by all indications, comparable (Shen *et al.*, 2023).

In conclusion, we assert the integrity of our study within the confines of its retrospective design, while acknowledging the inherent limitations in addressing every potential confounder that could connote a poor prognosis, a term that itself carries multifaceted definitions.
